# Cryptic genomic imbalances in patients with *de novo *or familial apparently balanced translocations and abnormal phenotype

**DOI:** 10.1186/1755-8166-1-15

**Published:** 2008-07-21

**Authors:** Carolina Sismani, Sofia Kitsiou-Tzeli, Marios Ioannides, Christodoulos Christodoulou, Violetta Anastasiadou, Goula Stylianidou, Eleftheria Papadopoulou, Emanuel Kanavakis, Zoe Kosmaidou-Aravidou, Philippos C Patsalis

**Affiliations:** 1 Department of Cytogenetics, The Cyprus Institute of Neurology and Genetics Nicosia, Cyprus; 2 Department of Medical Genetics, University of Athens, Choremio Research Laboratory, "Aghia Sophia" Children's Hospital, Athens, Greece; 3 Department of Pediatrics, Arch Makarios III Hospital, Nicosia, Cyprus; 4 Department of Paediatrics, University Hospital of Heraklion, Crete, Greece; 5 Department of Genetics, Alexandra Hospital, Athens, Greece

## Abstract

**Background:**

Carriers of apparently balanced translocations are usually phenotypically normal; however in about 6% of *de novo *cases, an abnormal phenotype is present. In the current study we investigated 12 patients, six *de novo *and six familial, with apparently balanced translocations and mental retardation and/or congenital malformations by applying 1 Mb resolution array-CGH. In all *de novo *cases, only the patient was a carrier of the translocation and had abnormal phenotype. In five out of the six familial cases, the phenotype of the patient was abnormal, although the karyotype appeared identical to other phenotypically normal carriers of the family. In the sixth familial case, all carriers of the translocations had an abnormal phenotype.

**Results:**

Chromosomal and FISH analyses suggested that the rearrangements were "truly balanced" in all patients. However, array-CGH, revealed cryptic imbalances in three cases (3/12, 25%), two *de novo *(2/12, 33.3%) and one familial (1/12, 16.6%). The nature and type of abnormalities differed among the cases. In the first case, what was identified as a *de novo *t(9;15)(q31;q26.1), a complex rearrangement was revealed involving a ~6.1 Mb duplication on the long arm of chromosome 9, an ~10 Mb deletion and an inversion both on the long arm of chromosome 15. These imbalances were located near the translocation breakpoints. In the second case of a *de novo *t(4;9)(q25;q21.2), an ~6.6 Mb deletion was identified on the short arm of chromosome 7 which is unrelated to the translocation. In the third case, of a familial, t(4;7)(q13.3;p15.3), two deletions of ~4.3 Mb and ~2.3 Mb were found, each at one of the two translocation breakpoints. In the remaining cases the translocations appeared balanced at 1 Mb resolution.

**Conclusion:**

This study investigated both *de novo *and familial apparently balanced translocations unlike other relatively large studies which are mainly focused on *de novo *cases. This study provides additional evidence that cryptic genomic imbalances are common in patients with abnormal phenotype and "apparently balanced" translocations not only in *de novo *but can also occur in familial cases. The use of microarrays with higher resolution such as oligo-arrays may reveal that the frequency of cryptic genomic imbalances among these patients is higher.

## Background

The great majority of cases with apparently balanced translocations are usually not associated with abnormal phenotypes. Apparently balanced rearrangements in general, represent an interpretational and counseling dilemma when detected in cases with abnormal phenotypes and/or mental retardation.

The risk for phenotypic abnormalities differs between *de novo *and familial balanced translocations. Warburton et al., 1991 [1] estimated the risk in carriers of *de novo *reciprocal translocations detected at prenatal diagnosis to be 6,1%, while Madan et al., 1997 [2] in a subsequent review of 60 cases revealed that the risk is even higher when the translocation is complex, as 14/27 of the complex cases were associated with multiple congenital anomalies and/or mental retardation.

In familial cases, the increased risk lies mainly in the production of abnormal gametes which leads to multiple miscarriages or to the birth of a child with congenital abnormalities. If the same balanced rearrangement as in the carrier parent is detected at prenatal diagnosis, the risk for phenotypic abnormality in the offspring is believed to be very low. However, there are several studies that report patients with abnormal phenotype and the same balanced rearrangement as their phenotypically normal carrier parent [3-5].

Several mechanisms were proposed to explain the clinical phenotype in both *de novo *and familial balanced translocations, which includes :i) disruption of a dosage-sensitive gene at the breakpoints or expression a recessive gene [6], ii) position effect with variable expression of genes near the translocation breakpoint [7] iii) uniparental disomy (if the chromosome involved is subjected to imprinting) due to post-conceptional "correcting" loss of the homolog from the normal non-carrier parent [8] iv) the rearrangement is not truly balanced at the DNA level or in familial cases may be additional unbalanced subtle rearrangements occurred during meiosis [9] v) the rearrangement may host 'cryptic' complex chromosomal rearrangements (CCRs)[10].

One of the first studies that investigated the possibility that apparently balanced translocations may in fact be CCRs was carried out by our group in 2004 [10] and 20 families (18 inherited and 2 *de novo*) were analyzed. The above study detected CCRs in 3 out of the 20 families studied and suggested that the link between an apparently balanced rearrangement and the appearance of abnormal phenotype may be partly explained by the presence of cryptic complex chromosomal rearrangements and that more breaks may lead to imbalances. The above study was based only on FISH approaches and therefore small interstitial rearrangements/imbalances may have been missed. The development of the array-based comparative genomic hybridization (array-CGH) overcame the limitations of both cytogenetic and FISH approaches and provided the opportunity to screen the entire genome for cryptic genomic gains and losses [5,11,12].

A high level of unexpected rearrangement complexity, including deletions, inversions and insertions at or near one or both breakpoints as well as imbalances on chromosomes unrelated to the translocations were found by Gribble et al., 2005 [13] in their systematic analysis of constitutional *de novo *apparently balanced carriers. Imbalances were detected in 6/10 (60%) of the patients studied.

Most of the recent relatively large studies are however focused on *de novo *balanced translocations [13,14]. In the current study, we report 12 cases, six *de novo *and six familial, of apparently two-way balanced translocations in patients with mental retardation and congenital malformations that were investigated by 1 Mb array-CGH. In the familial cases the patients had an abnormal phenotype but their karyotype appeared identical to other phenotypically normal translocation carriers of the family. In one familial case all the carriers of the translocations had abnormal phenotype.

## Results

Array-CGH revealed cryptic genomic imbalances in three cases out of the twelve cases studied (25%), two out of the six *de novo *(33.3%) and one out of the six familial cases (16.6%). Summarized results of all the aberrations detected by array-CGH are shown in table 1. The imbalances detected by array-CGH among the *de novo *cases were not found in their chromosomally and phenotypically normal parents thus excluding polymorphism.

**Table 1:** shows the abnormalities detected by array-CGH.

**Case**	**Initial Karyotype**	**Imbalances (array-CGH)**	**Size**
**1**	46,XY,t(9;15)(q31;q26.1)de novo	dup(9)(q34.1q34.3)	~6.1 Mb
		del(15)-complex	~10 Mb
		inv(15)(q21.1q22.3)	~3 Mb
**2**	46,XY,t(4;9)(q25;q21.2)de novo	del(7)(p12.3p13)	~6.6 Mb
**3**	46,XX,t(4;7)(q13.3;p15.3)mat	del(4)(q13.3q13.3)	~4.3 Mb
		del(7)(p15.3p21.1)	~2.3 Mb

In five out of the six familial cases the patients of each family had an abnormal phenotype but their karyotype appeared identical to other phenotypically normal translocation carriers of the family. No imbalances were detected in these five cases and the translocation appeared balanced at 1 Mb resolution.

In one of the six familial cases, all the carriers of the translocations in the family including the patient's mother and sister shared the same abnormal phenotype. Array-CGH revealed the same imbalances that were initially detected in the older daughter, in her mother and sister.

### Case 1: 46,XY,t(9;15)(q31;q26.1)de novo

The above patient is a 2-year-old boy with a peculiar facies and psychomotor retardation, especially gross motor retardation and speech delay. His weight is 11.700 gr (25^th ^centile), the height 86 cm (50^th ^centile), and the head circumference 49 cm (75^th ^centile). He was born by a caesarean section at 35 weeks' gestation with a small weight for gestational age (1.880 gr, <3^rd ^centile). As an infant the patient presented hypotonia in association with severe recurrent episodes of apnea, bradycardia and cyanosis, almost on a daily base. Patient's dysmorphic features include frontal bossing with narrow biparietal diameter, prominent eyes with blue sclerae, broad nasal bridge, low set ears, high narrow palate, retrognathia, upsweep of frontal scalp hair, abnormal palmar creases, bilateral overlapping toes, and omphalocele. In addition, the patient has gastro-esophageal reflux, a small atrial septal defect, and a right renal rotation around its elongated axis.

**Figure 1:**
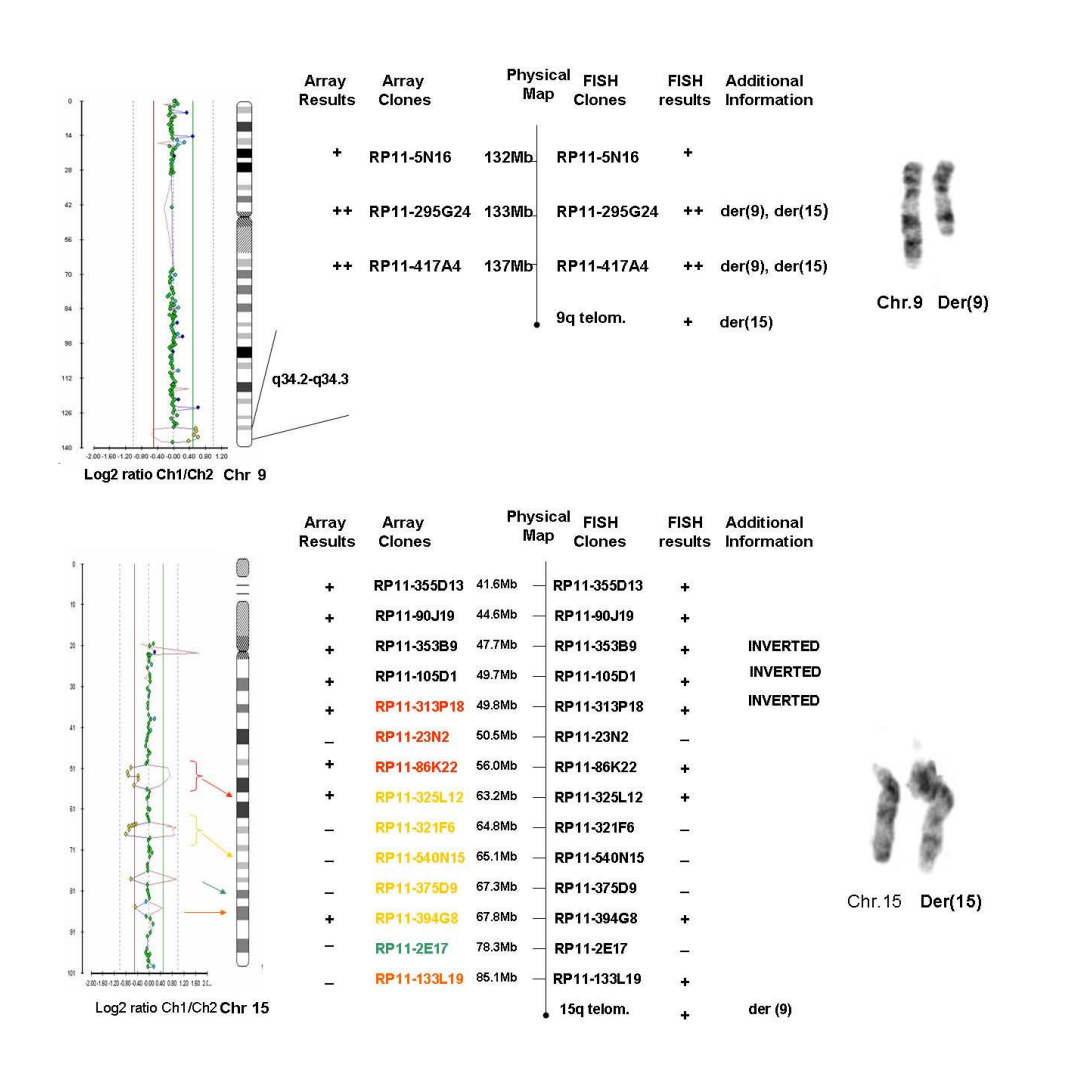
Array-CGH results of case 1 for chromosomes 9 and 15, schematic comparison of the array and FISH findings and partial karyotypes.

Chromosomal and FISH [subtelomeric and whole chromosome paint (wcp)] analyses of this patient showed an apparently balanced de novo translocation between chromosomes 9 and 15. The initial karyotype was designated as 46,XY,t(9;15)(q31;q26.1)dn. Array-CGH though revealed an ~6.1 Mb duplication on chromosome 9 at 9q34.2-q34.3 and deletions on three regions of chromosome 15 adding to ~10 Mb (Figure 1). A fourth region also appeared deleted on chromosome 15 but FISH analysis using the corresponding BAC clone showed normal signals. The imbalances detected by array-CGH were on the same chromosomes involved in the translocation but not directly associated with them. The array-CGH findings were confirmed using FISH with 14 region-specific BAC clones. During confirmation of the array findings, the rearrangement was found to be even more complex with additional breakpoints (at least 5 breakpoints) and an inversion was unexpectedly identified involving chromosome 15 with breakpoints at q21.1 and q21.2. FISH using the same BAC clones was also performed for the parents of the patients and normal results were obtained.

Revised karyotype after FISH confirmation of the array results:

46,XY,t(9;15)(q31;q26.1).ish dup(9)(q34.1q34.3)(RP11-295G24++, RP11-417A4++),del(15)(q21.2q21.3)(RP11-32N2-),del(15)(q22.31q23)(RP11-321F6-, RP11-540N15-, RP11-375D9-),del(15)(q25.1q25.2)(RP11-2E17-), inv(15)(q21.1q21.2)(RP11-90J19st,RP11-353B9mv,RP11-105D1mv, RP11-313P18mv)dn.

### Case 2: 46,XY,t(4;9)(q25;q21.2)de novo

The patient, a 22 years old male, has a history of mild mental and growth retardation, motor dyspraxia and myoclonic seizures. He presents antimongoloid palpebral fissures, microtia, high-arched palate, low-set and dysplastic ears, strabismus, bulbous nose, short webbed neck, low posterior hairline, scoliosis, lordosis, short nails and broad first toes bilaterally.

**Figure 2:**
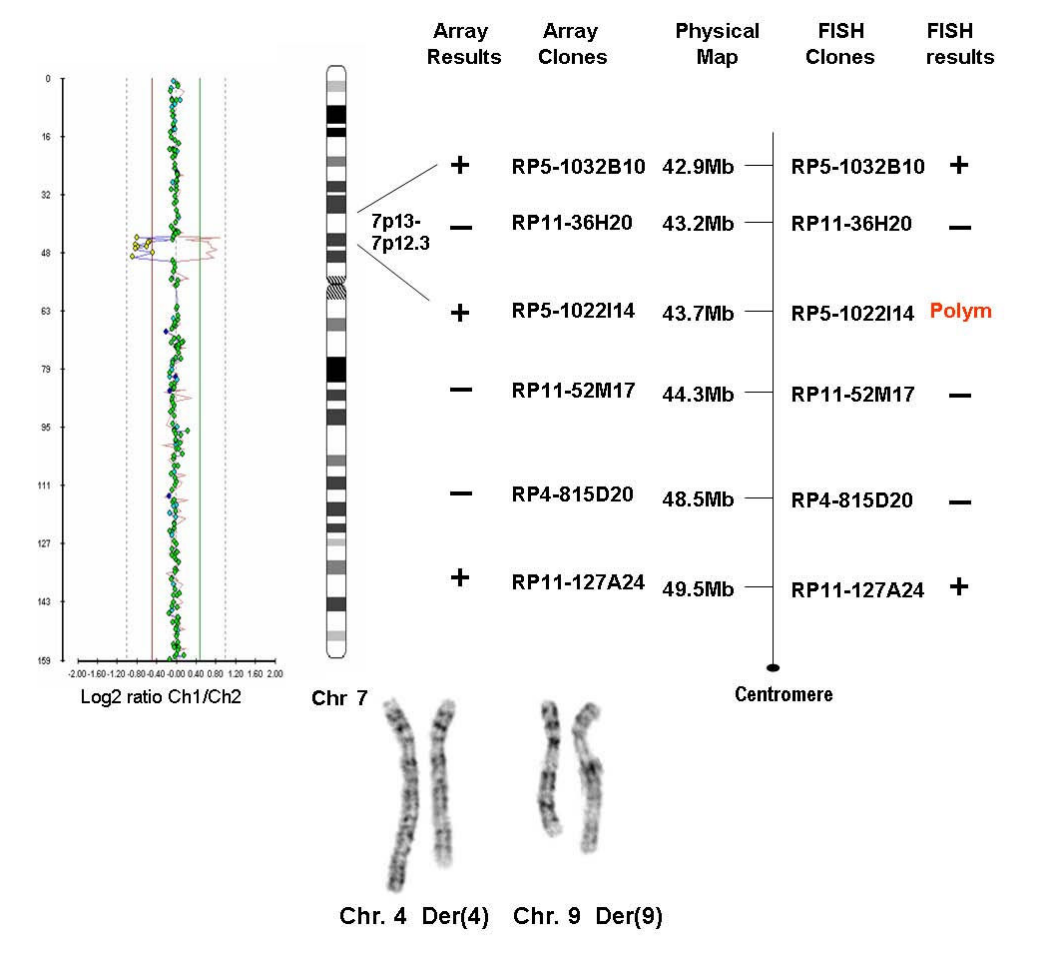
Array-CGH results of case 2 for chromosome 7 and schematic comparison of the array and FISH findings and partial karyotypes.

Chromosomal and FISH (subtelomeric and wcp) analyses of this patient showed an apparently balanced *de novo *translocation between chromosomes 4 and 9. The initial karyotype was designated as 46,XY,t(4;9)(q25;q21.2)dn. Array-CGH however showed an ~6.6 Mb deletion on chromosome 7 which is unrelated to the translocation (Figure 2). The above findings were confirmed using FISH with 6 region-specific BAC clones.

Revised karyotype after FISH confirmation of the array results:

46,XY,t(4;9)(q25;q21.2).ish del(7)(p12.3p13)(RP4-815D20-,RP11-52M17,RP11-36H20-)dn.

### Case 3: 46,XX,t(4;7)(q13.3;p15.3)mat

Case 3 involves a familial translocation between chromosomes 4 and 7 which was inherited from the mother. The above family has 3 children, two daughters that are both carriers of the translocation and share the same clinical phenotype and one son with normal karyotype and phenotype. The older daughter is currently 16 years old and the younger is 15. They were both born after uneventful pregnancies and deliveries. As infants, they had feeding problems and delayed motor milestones. They both have moderate mental retardation, seizures, attention deficit hyperactivity syndrome and severe learning difficulties. They attend school for children with special needs. They have no dysmorphic features or other congenital abnormalities. The mother has also mental retardation but in milder form.

**Figure 3:**
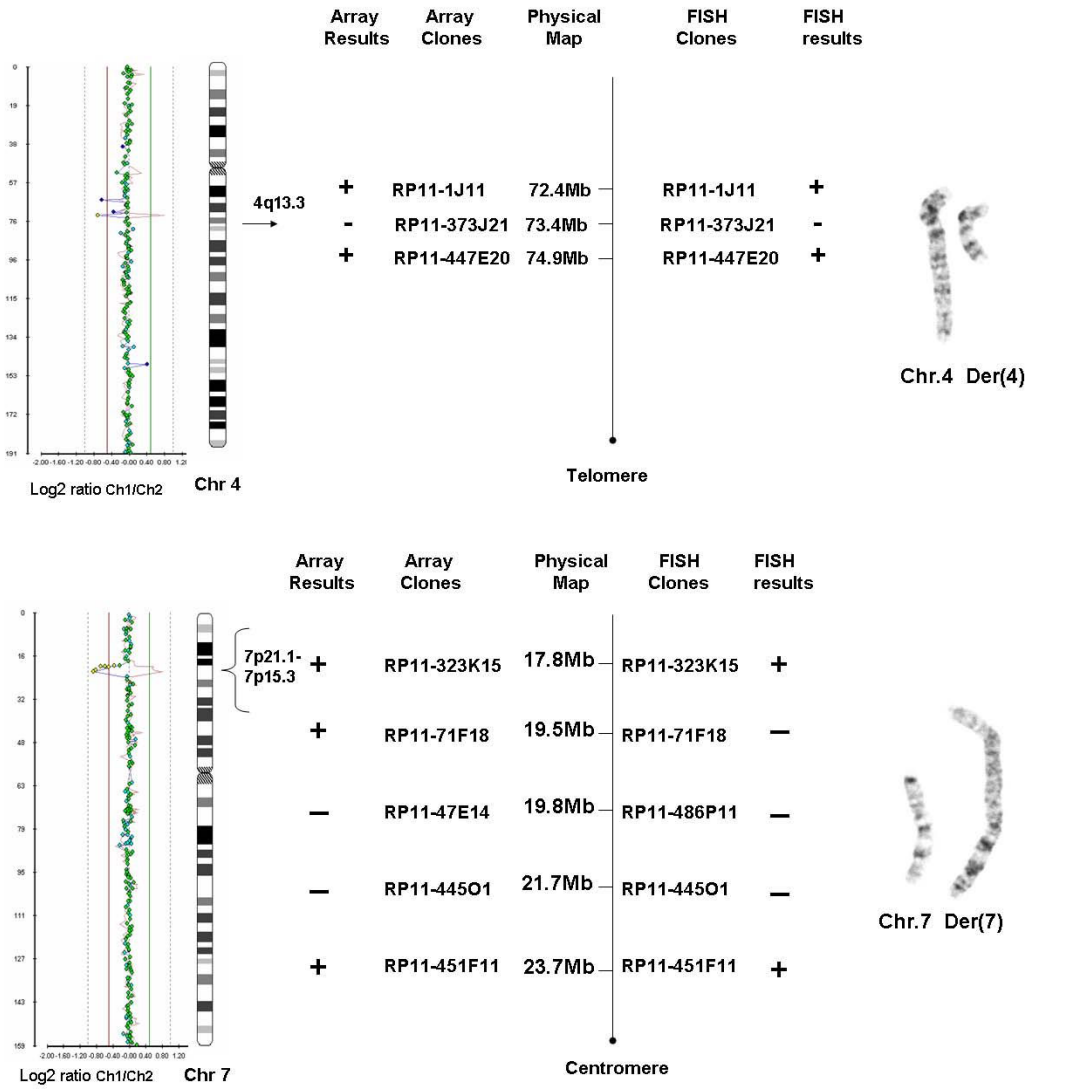
Array-CGH results of case 3 for chromosomes 4 and 7 and schematic comparison of the array and FISH findings and partial karyotypes.

Chromosomal and FISH (subtelomeric and wcp probes) analyses of the mother and her daughters revealed apparently identical karyotypes: 46,XX,t(4;7)(q13.3;p15.3). Array-CGH revealed an ~4.3 Mb and an ~2.3 Mb deletion on chromosome 4 and 7, respectively in all carriers (Figure 3). These copy number changes were located at the breakpoints of the translocations and were confirmed with 6 region-specific BAC clones.

Revised karyotype after FISH confirmation of the array results:

46,XX,t(4;7)(q13.3;p15.3).ish del(4)(q13.3q13.3)(RP11-373J21-), del(7)(p15.3p21.1)(RP11-445O1-,RP11-47E14-)mat.

## Discussion

Array-CGH revealed cryptic genomic imbalances at 1 Mb resolution in three out of the twelve cases studied (25%) with an apparently balanced translocation and abnormal phenotype, in two of the six *de novo *cases (33.3%) and in one of the six familial cases (16.6%). The remaining of the cases appeared to be simple and balanced at 1 Mb resolution.

The present study is to our knowledge the first where a relatively large number of patients with familial balanced translocations and abnormal phenotype were investigated for cryptic imbalances using array-CGH, unlike most of the studies that are focused on *de novo *cases. Usually, familial cases are reported as individual case studies. Only one other study has been performed with array-CGH by [5]Ciccone et al.,2005 in four cases, two *de novo *and two familial of patients with balanced translocations and abnormal phenotype.

The frequency of imbalances detected in the *de novo *cases is in accordance with the largest systematic study performed to date by De Gregori et al 2007 [14], where 27 apparently balanced de novo translocations with abnormal clinical phenotype were analyzed with high density (20 kb or 100 kb resolution) oligo array-CGH and 11 cases with copy number imbalances (40.7%) were detected. Another study by Gribble et al, 2005, [13] using 1 Mb resolution array-CGH, detected imbalances in 6 out of 10 (60%) of the *de novo *balanced translocation cases with abnormal phenotype. Although the sample sizes are still small, we can safely assume by combining the two previous studies with the supporting evidence of the current study that about 30–50% of the *de novo *apparently balanced translocations with abnormal phenotype are associated with causative cryptic imbalances. In the remaining 50–70% of the patients the phenotype might me caused by other mechanisms such as interruption of a dosage sensitive gene, or by uniparental disomy or by unmasking of a recessive mutated gene on the homolog chromosome or finally by position effect with variable expression of a gene(s) near the translocation breakpoint. However a disruption of a recessive gene might occur at the breakpoints of the translocation without a phenotypic effect. Baptista et al., 2008 [15] studied 31 cases of balanced translocation carriers with normal phenotype using FISH to refine the breakpoints and 1 Mb array-CGH to detect imbalances. In this study no cryptic imbalanced were detected in individuals with normal phenotype but interruption of genes was detected in 16 of the 31 individuals studied.

Only in one case (Case 3), out of the six familial cases analyzed, additional aberrations were identified, which involved a family where all translocation carriers shared the same abnormal phenotype. The deletions detected at the breakpoints of the translocation were present in all carrier family members and absent in the phenotypically normal members of the family. In the remaining five families in which only the patients had an abnormal phenotype while all the other carriers were phenotypically normal no additional aberrations were detected at the resolution of 1 Mb. Results obtained in the current study from the familial cases suggest that cryptic copy number changes at least at the resolution of 1 Mb, do not constitute a major cause for the phenotypic abnormalities in patients with familial apparently balanced translocations. However, the study by Ciccone et al 2005 [5], revealed cryptic imbalances in one *de novo *case and in both patients with familial translocations suggesting that cryptic imbalances in familial cases may be common. Therefore more familial cases need to be analyzed in order to draw final conclusions. It is possible that higher resolution arrays such as high-density oligo-arrays may reveal smaller aberration that could have been missed in the current study due to lower resolution. It is also possible, that other mechanism are involved in inherited cases like production of a recessive mutation by the translocation that is expressed when a mutation in the same gene is inherited from the other parent or the phenotype might simply be coincidental and unrelated to the translocation.

It is interesting that in a study published by our group in 2004 [10], in which balanced translocations were investigated for the presence of cryptic complex rearrangements, case 3 was then was classified as simple since only FISH approaches were used. As a result the imbalances detected here by array-CGH were missed. This demonstrates the value of array-CGH for the detection of cryptic subtle rearrangements. However, it is also important to point out that confirmation of array-CGH results with FISH studies is always necessary, as it was illustrated in Case1 of the present study, where during FISH confirmation, an inversion was unexpectedly revealed.

The nature, location and size of the abnormalities differed among the three cases. Among the imbalances detected three were deletions, one was a duplication and one an inversion and their size ranged from 2.3 to 10 Mb. The locations of these rearrangements relative to the translocation breakpoints were also different from case to case. In the first case, the imbalances were on the same chromosomes involved in the translocation but were not directly associated with the breakpoints, in the second case a deletion was identified on a chromosome, unrelated to the translocation and in the third case imbalances were found at the translocation breakpoints. The above results show that cryptic aberrations are not always related to the rearrangements breakpoints. Similar results were previously reported by other studies [5,13,14].

Balanced translocations remain a challenge for geneticists especially when they are detected prenatally. In the case of familial translocations, the translocation present in the parent should always be investigated with FISH assays to determine whether it is a simple two way translocation or it involves more chromosomes so that more accurate diagnosis in the fetus is performed. In *de novo *cases, it should also be investigated with FISH assays since more breaks usually involve higher risk. In addition, in cases of *de novo *translocations, array-CGH should be performed as subtle copy number changes may be present not only at the breakpoints but anywhere else in the genome. Although, array-CGH is well established method and is widely used for the evaluation of patients with mental retardation and congenital abnormalities, its implementation in prenatal diagnosis is still very limited. A major factor for the limited used of array-CGH in prenatal diagnosis is the presence of benign copy number variations (CNVs) [16,17] which can extend up to several Mb and may cause problems in result interpretation. However, as new publications and databases with CNV data emerge, array CGH will be incorporated in the methodologies used in prenatal diagnosis, especially in cases with normal or "balanced karyotypes" and ultrasound abnormalities.

## Conclusion

The current study provides additional evidence and confirms previous studies which showed that cryptic genomic imbalances are common (33.3%) in patients with abnormal phenotype and *de novo *"apparently balanced" translocations. In contrast to previous investigations both *de novo *(six cases) and familial (six cases) cases were analyzed by array-CGH and imbalances were identified in both *de novo *and familial cases. These imbalances were either linked (near or at the breakpoints of the translocation) or were unrelated to the rearrangement. These findings further highlight the necessity of whole genome approaches for "apparently balanced" rearrangements.

## Methods

### Patients

Twelve patients with apparently balanced translocations familial or de novo and abnormal clinical phenotype were included in the study. In all *de novo *cases, only one member of each family was a carrier of a translocation and had an abnormal phenotype while all the other members had normal karyotypes and normal phenotypes. In five out of the six familial cases the patients of each family had an abnormal phenotype but their karyotype appeared identical to other phenotypically normal translocation carriers of the family. In one familial case, all the carriers of the translocations had abnormal phenotype.

Family history and clinical information of each patient were recorded. Patients were included in the study only if chromosomal and FISH analyses suggested that the rearrangement was "truly" balanced.

### Chromosomal and FISH Studies

Chromosomal analyses were performed from peripheral blood samples using conventional GTG-banding techniques at the 550-band level.

FISH analyses were performed using commercially available subtelomeric specific probes and whole chromosome paints (wcp) to confirm that the translocations were "truly balanced". FISH procedures were performed according to the manufacturer's protocols (VYSIS. Inc, USA).

FISH was also carried out using BAC-probes for confirmations of the array-CGH findings. Location of the clones was obtained from NCBI . Labelling and FISH procedures were performed according to the manufacturer's protocols (VYSIS. Inc, USA)

### Array-CGH

DNA was isolated using the PUREGENE DNA extraction kit (Gentra, USA) according to the supplier's protocol. The Cytochip 1-Mb resolution chip (BlueGnome, Cambridge, UK) was used for microarray-CGH analysis. Array-CGH was carried out according to the recommendations of the manufacturer. In brief, DNAs were labelled by random priming using Bio Prime labelling kit (Invitrogen, UK) with cyanine 3 and cyanine 5 (Amersham Biosciences, UK) fluorescent dyes. DNAs were co-hybridized on two microarrays as dye swap was used. Pooled genomic DNA from Promega (UK) was used as reference. Data was analysed with the BlueFuse for microarrays software package (BlueGnome, Cambridge, UK).

## Authors' contributions

CS drafted the manuscript and carried out the array-CGH assays, MI and CC performed the FISH assays SKT, VA, GS, EP, ZKA and EK performed the clinical evaluation of the patients, PP conceived the study, participated in its design and coordination and also approved the manuscript.
